# Fabrication and *in vitro *deployment of a laser-activated shape memory polymer vascular stent

**DOI:** 10.1186/1475-925X-6-43

**Published:** 2007-11-27

**Authors:** Géraldine M Baer, Ward Small, Thomas S Wilson, William J Benett, Dennis L Matthews, Jonathan Hartman, Duncan J Maitland

**Affiliations:** 1 Department of Biomedical Engineering, University of California, Davis, California 95616 USA; 2 Lawrence Livermore National Laboratory, Livermore, California 94550 USA; 3 Department of Applied Science and School of Medicine, University of California, Davis, California 95616 USA; 4 Kaiser Permanente Medical Center, Sacramento, CA 95825 USA and Department of Radiology, University of California, Davis, School of Medicine, Sacramento, CA 95817 USA

## Abstract

**Background:**

Vascular stents are small tubular scaffolds used in the treatment of arterial stenosis (narrowing of the vessel). Most vascular stents are metallic and are deployed either by balloon expansion or by self-expansion. A shape memory polymer (SMP) stent may enhance flexibility, compliance, and drug elution compared to its current metallic counterparts. The purpose of this study was to describe the fabrication of a laser-activated SMP stent and demonstrate photothermal expansion of the stent in an *in vitro *artery model.

**Methods:**

A novel SMP stent was fabricated from thermoplastic polyurethane. A solid SMP tube formed by dip coating a stainless steel pin was laser-etched to create the mesh pattern of the finished stent. The stent was crimped over a fiber-optic cylindrical light diffuser coupled to an infrared diode laser. Photothermal actuation of the stent was performed in a water-filled mock artery.

**Results:**

At a physiological flow rate, the stent did not fully expand at the maximum laser power (8.6 W) due to convective cooling. However, under zero flow, simulating the technique of endovascular flow occlusion, complete laser actuation was achieved in the mock artery at a laser power of ~8 W.

**Conclusion:**

We have shown the design and fabrication of an SMP stent and a means of light delivery for photothermal actuation. Though further studies are required to optimize the device and assess thermal tissue damage, photothermal actuation of the SMP stent was demonstrated.

## Background

Vascular stents are small tubular scaffolds used to maintain the luminal size of an artery. They are now widely used in conjunction with transluminal angioplasty in the treatment of arterial stenosis (narrowing of the vessel) to prevent acute vessel closure and late restenosis in a variety of large vessels such as coronary arteries [1], carotid arteries [2], and iliac arteries [3]. Most stents are currently made of stainless steel, shape memory alloys (SMAs), and other metal alloys. Crimped over a catheter, they are navigated to the lesion site where they are expanded either by balloon expansion or by self-expansion. Though improvements in design and coating with drug-eluting agents have resulted in smaller, safer, and more biocompatible stents with reduced rates of restenosis [4], several drawbacks associated with the use of metallic stents still exist. First, metallic stents are too stiff to navigate highly tortuous vessels such as those of the neurovasculature; the need for treatment of stroke and intracranial stenosis is well known [5], but successful stenting with the current technology has been shown in a limited number of cases only [6]. Second, compliance mismatch due to the stiffness of metallic stents at the arterial wall may potentially be a contributing factor in restenosis based on trapped proliferating cells observed for a stent with compliance-matched ends [7]. Third, drug elution is currently achieved by coating the metal with a drug-doped polymer [8], which requires a costly additional fabrication step [9].

Thermally activated shape memory polymer (SMP) is a unique class of polymeric materials that can maintain a secondary shape and then recover a pre-determined primary shape when sufficiently heated. SMP has been shown to have promising applications in intravascular medical devices for thrombectomy [10,11], aneurysm embolization [12,13], and as vascular stents [14]. Our previous investigation of the thermomechanical properties of shape memory polyurethane revealed high recovery strains, which are needed for large shape changes during stent deployment [15]. In addition, the glassy modulus of SMP is lower than those of SMAs and stainless steel by a factor of at least 10 and 100, respectively, potentially yielding a more flexible and compliant structure depending on the amount of material and the stent design pattern [16]. Ongoing biocompatibility studies have shown that the material has minimal effect on triggering an inflammatory response, activating platelets and neutrophils, and inducing thrombogenesis *in vitro *[17]. Finally, drug embedding in polymer stents has already been demonstrated [18]. Thus, the large strain recovery, flexibility, conformability, and compliance of SMPs could overcome the problems encountered with current metallic vascular stents.

Light absorption has been shown to be an effective heating mechanism for actuating SMPs [10,11,19,20]. In this paper, we present a photothermally activated self-expanding stent made of shape memory polyurethane. The SMP stent is crimped over a light diffuser attached to the end of an optical fiber coupled to an infrared diode laser. The optical fiber serves as both the transport vehicle and the conduit for light energy delivery to the stent. We describe the design and fabrication of the device and demonstrate laser actuation in a water-filled *in vitro *artery model.

## Methods

### Shape memory polymer

The SMP used in this study was MM5520 thermoplastic polyurethane; it was obtained from DiAPLEX Company, Ltd. (a subsidiary of Mitsubishi Heavy Industries Ltd., Tokyo, Japan) in the form of pellets. The composition is proprietary, but it is known that the material is a segmented polyurethane with a microphase separated morphology consisting of a hard phase and a soft phase [21]. The nominal temperature of the soft phase glass transition (T_g_) was 55°C. The shape memory effect comes from the entropic potential for recovery of the primary shape, stored in macromolecular chains of the soft phase in the secondary shape. The primary shape can be formed by heating the material above the hard phase glass transition temperature, deforming the material into the desired shape, and then cooling to fix the shape. Alternatively, the material may be dissolved in a suitable solvent and cast in a mold or coated onto a surface (as was done in this study) and vacuum dried to establish the primary shape. The secondary shape is obtained by heating the material above T_g_, deforming the material (in this case crimping), and cooling to fix the shape. Heating the material again to T_g _results in recovery of the primary shape. Due to the broad soft phase glass transition of MM5520 [11], shape recovery begins to occur at 40–45°C (10–15°C below the nominal T_g _of 55°C). The material exhibits different mechanical properties above and below the T_g_. Our previous studies of this specific polymer have shown a glassy modulus of ~800 MPa (shear modulus at T_g_-25°C), and a rubbery modulus of ~1.4 MPa (shear modulus at T_g _+ 25°C) [15]. The glassy modulus of this SMP (~800 MPa) lies in the range of reported values for balloon-expandable polymer stents, which starts at 400 MPa (for applications requiring low mechanical strength, such as neural stenting) and extends up to 7 GPa [22]. Accordingly, the relatively low glassy modulus (and, hence, low expansion force) of the SMP stent may require balloon angioplasty prior to laser expansion.

### Device fabrication

**Figure 1:**
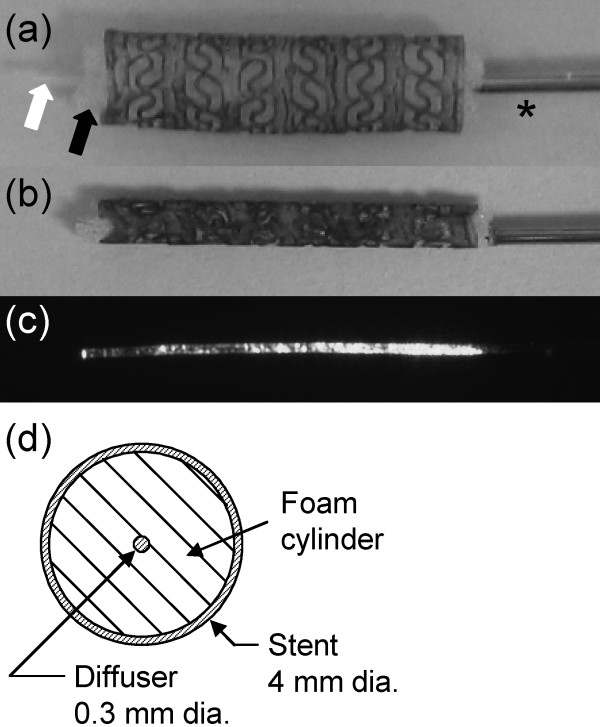
**SMP stent**. Close-up of the SMP stent mounted over the SMP foam cylinder (black arrow) and the coaxial SMP diffuser (white arrow) prior to crimping (a) and after crimping (b). The distal end of the stainless steel hypotube containing the optical fiber is indicated by the asterisk in (a). An image of the diffuser emission, which decreases with distance from the optical fiber tip, is shown in (c). A cross-sectional diagram of the expanded stent with the diffuser and foam cylinder in place is shown in (d).

Fabrication of the stent began by dissolving pellets of MM5520 in tetrahydrofuran (THF) to prepare a solution with a composition of 17.5% by weight of polymer in solvent. A SMP tube was first made by dip coating a 4 mm diameter stainless steel pin in the solution multiple times at a constant rate of withdrawal. The coated pin was then dried for 24 hours at 50°C under vacuum. After cooling, the SMP tube was laser etched to impart a specific pattern based on an imported CAD file using a computer-controlled system. As shown in Fig. 1(a), solid rings are connected by S-shaped struts, enhancing its longitudinal flexibility. The stent was then removed from the pin using hot water, dried, doped with a laser-absorbing platinum dye (Epolight 4121, Epolin, Inc.), and dried again for 24 hours at 50°C under vacuum. The dye was incorporated by coating the stent with a solution of 4440 ppm dye in THF. The stent diameter decreased slightly after removal from the pin due to residual stress in the SMP. The final stent in its expanded state was 4.1 mm outer diameter by 16 mm long with a wall thickness of 250 μm.

The light delivery/transport vehicle assembly consisted of two main components, both of which were fabricated using SMP: (1) a 300 μm diameter by ~16 mm long cylindrical light diffusing SMP rod attached to the end of an optical fiber and (2) a coaxial 5 mm diameter by ~16 mm long SMP foam cylinder surrounding the diffusing rod. This assembly was inserted inside the stent lumen prior to crimping (Fig. 1(a,c)). The diffuser was fabricated by casting an SMP formulation developed in-house [23] over the distal end of a 100 μm core diameter silica optical fiber (FIP100110125, Polymicro Technologies, LLC) using a tubular mold made of Teflon. To enable light diffusion, the surface of the cured SMP cylinder was abraded using a media blaster loaded with sodium bicarbonate powder. The longitudinal emission profile of the diffuser, acquired using a CCD camera with the optical fiber coupled to a red 4.2 mW diode laser, is shown in Fig. 1(d). Approximately 80% of the incident laser light was emitted in the first centimeter of the diffuser. The optical fiber was sheathed with a 711 μm outer diameter stainless steel hypotube to provide a pushable transport vehicle.

The foam component was made of in-house formulated SMP material consisting of hexamethylene diisocyanate (HDI), N,N,N'-tetrakis(2-hydroxypropyl)ethylenediamine (HPED), and triethanolamine (TEA) [13]. The density of the foam was 0.020 g/cm^3 ^with mostly open cells approximately 200 μm in size (determined by optical microscopy). The calculated volumetric void fraction was 98.4%, which would allow a theoretical expansion of 60 times from the fully condensed state. Differential scanning calorimetry measurements showed a T_g _around 40°C. The foam cylinder was cut using a 5 mm biopsy punch and then slightly compressed under warm air to enable it to fit inside the expanded stent. The role of the foam cylinder is to assist photothermal expansion of the device by (1) centering the diffuser in the stent lumen, (2) increasing laser light scattering to improve illumination uniformity, and (3) reducing convective cooling of the stent. Though not necessary in this initial prototype, the foam would be bonded to the diffuser to facilitate withdrawal after stent deployment in a clinical device.

With the diffuser/foam assembly positioned inside the stent lumen as shown in Fig. 1(a,c), the final device was mechanically compressed using a crimping machine (Balloon Wrapping Fixture Model W8FH, Interface Associates, Inc.) from 4.1 mm down to 1.8 mm (smaller diameters are possible depending on the amount of material and stent design). The machine consists of eight radially arranged heated blades which form a cylindrical cavity; as the blades move inward, the cavity diameter decreases. The blades were heated to 88°C prior to and during compression, and then cooled to room temperature before the crimped device was removed. The device remained in the crimped shape before experimental actuation. Fig. 1(b) shows the device in its crimped state.

### Artery model and device actuation

**Figure 2:**
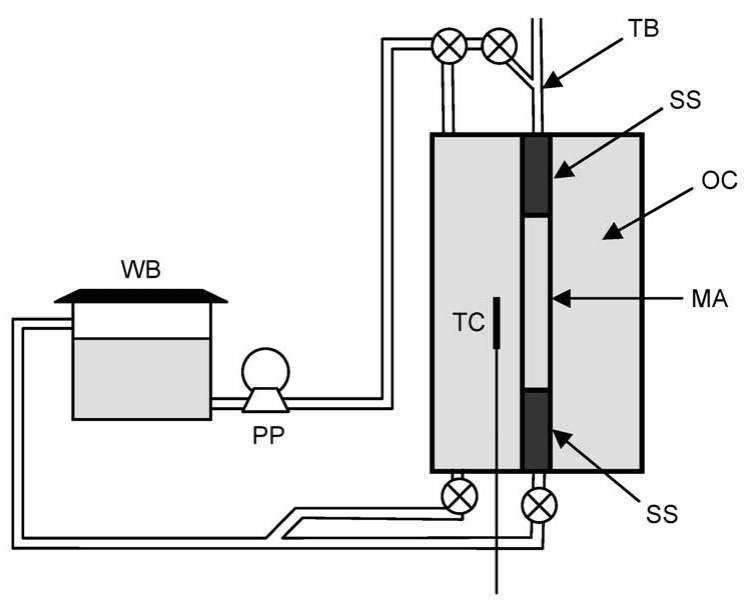
**Artery model**. Schematic of the artery model. WB: water bath, PP: peristaltic pump, TB: Touhy Borst valve, SS: stainless steel cylinder, OC: outer chamber, TC: thermocouple probe, MA: mock artery.

An artery model consisting of a straight mock artery in a chamber was built in-house. The mock artery (4 mm inner diameter, 150 μm wall thickness, 4 cm length) was made of a silicone elastomer (Sylgard 184, Dow Corning) prepared by dip coating pins in a similar manner as the stent. As shown in Fig. 2, two stainless steel cylinders hold the mock artery in place inside the chamber; water at a desired temperature can be pumped independently in the outer chamber and into the mock artery. A thermocouple probe allows temperature reading in the outer chamber. A Touhy Borst valve allows flow of water and delivery of the device into the mock artery. Water temperature in the outer chamber and in the mock artery was maintained at 37°C (body temperature). After coupling the optical fiber to an 810 nm diode laser (UM7800/100/20, Unique Mode), the device was delivered into the mock artery and photothermally actuated.

## Results

**Figure 3:**
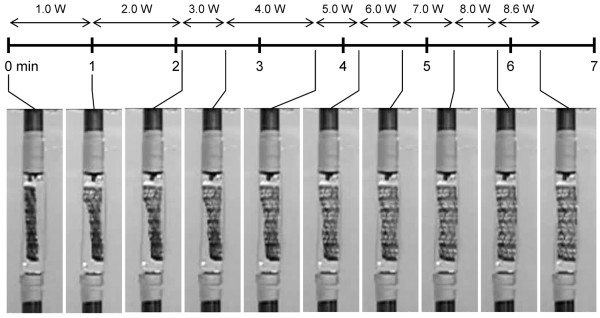
**Stent deployment**. Timeline of SMP stent deployment in the mock artery (zero flow) as the laser power was gradually increased. Laser duration was approximately 6.3 min.

After delivery to the mock artery, the stent did not show signs of expansion in the 37°C water for approximately 3 min prior to actuation. This indicates that the device would likely remain crimped during navigation and positioning under physiological conditions until it is controllably expanded, though further investigation is needed to confirm this hypothesis. Fig. 3 shows the device being actuated under zero flow as the laser power was slowly increased from 0 to 8.6 W in 1 W increments over 6.3 min (a time lapse video is provided in Additional file 1). The laser power could be increased more rapidly or kept constant in an optimized actuation protocol to reduce the actuation time. The end of the stent closer to the tip of the optical fiber rapidly expanded at a power of 1 W, consistent with the stronger proximal emission of the diffuser. The remainder of the stent fully expanded at ~8 W. The temperature inside the outer chamber did not vary during actuation, as indicated by the thermocouple probe. Following actuation, the diffuser was withdrawn and the stent and inner foam were retrieved from the mock artery by disassembling the chamber. The stent, foam, diffuser, and mock artery showed no visible signs of thermal damage. The experiment was repeated two more times with similar results.

In two separate attempts, the stent expanded only about 60% during actuation at 8.6 W for several minutes when the flow rate was increased to 180 ml/min, a value in the range of reported flows in the internal carotid artery [24]. This result suggests that the laser heating was overcome by convective cooling before full expansion was achieved.

## Discussion

In an actual clinical intervention, the inner foam would need to be withdrawn with the diffuser. As mentioned previously, one way to achieve this would be to bond the foam to the diffuser. Because the foam (T_g _~40°C) is compliant at body temperature, it can conform to the smaller diameter of the delivery catheter as it is pulled back for removal from the body. Tapering the proximal end of the foam may facilitate retraction back into the delivery catheter.

Due to the different physical properties of water and blood [25-30] (see Table 1), heat transfer between the laser-heated stent and the surrounding fluid is not necessarily identical for the two fluids. The difference was assessed for the two flow cases investigated: (1) zero flow and (2) physiological flow (180 ml/min). In the zero flow case, heat transfer occurs by conduction and natural convection. Because the thermal diffusivity of water is similar to that of blood, heat transfer by conduction is expected to be similar for the two fluids. Heat transfer by natural convection was assessed by estimating the Nusselt number (Nu), which represents the ratio of total heat transfer to conductive heat transfer. In the zero flow case for two concentric cylinders (collapsed stent centered in the mock artery), Nu for natural convection is given by [31]

**Table 1:** Physical properties of water and blood

Property	Water	Blood	Units
Density *ρ*	1000	1050 [25]	kg/m^3^
Specific heat *c*	4190 [26]	3850 [25]	J/(kg · K)
Thermal conductivity *k*	0.61 [27]	0.53 [28]	W/(m · K)
Thermal diffusivity *α *= *k*/*ρc*	1.4 × 10^-7^	1.3 × 10^-7^	m^2^/s
Absolute viscosity *μ*	7 × 10^-4 ^[26]	3.5 × 10^-3 ^[29]	Pa · s
Kinematic viscosity *ν *= *μ*/*ρ*	7 × 10^-7^	3.3 × 10^-6^	m^2^/s
Coefficient of thermal expansion *β*	2.1 × 10^-4 ^[26]	4.0 × 10^-4 ^[30]	K^-1^

Nu=0.603C¯lln⁡(Do/Di) Ra1/4[(L/Di)3/5+(L/Do)3/5]5/4
 MathType@MTEF@5@5@+=feaafiart1ev1aaatCvAUfKttLearuWrP9MDH5MBPbIqV92AaeXatLxBI9gBaebbnrfifHhDYfgasaacPC6xNi=xI8qiVKYPFjYdHaVhbbf9v8qqaqFr0xc9vqFj0dXdbba91qpepeI8k8fiI+fsY=rqGqVepae9pg0db9vqaiVgFr0xfr=xfr=xc9adbaqaaeGacaGaaiaabeqaaeqabiWaaaGcbaGaeeOta4KaeeyDauNaeyypa0JaeGimaaJaeiOla4IaeGOnayJaeGimaaJaeG4mamJafm4qamKbaebadaWgaaWcbaGaemiBaWgabeaajuaGdaWcaaqaaiGbcYgaSjabc6gaUnaabmaabaWaaSGbaeaacqWGebardaWgaaqaaiabd+gaVbqabaaabaGaemiraq0aaSbaaeaacqWGPbqAaeqaaaaaaiaawIcacaGLPaaacqqGGaaicqqGsbGucqqGHbqydaahaaqabeaacqqGXaqmcqqGVaWlcqqG0aanaaaabaWaamWaaeaadaqadaqaamaalyaabaGaemitaWeabaGaemiraq0aaSbaaeaacqWGPbqAaeqaaaaaaiaawIcacaGLPaaadaahaaqabeaacqaIZaWmcqGGVaWlcqaI1aqnaaGaey4kaSYaaeWaaeaadaWcgaqaaiabdYeambqaaiabdseaenaaBaaabaGaem4Ba8gabeaaaaaacaGLOaGaayzkaaWaaWbaaeqabaGaeG4mamJaei4la8IaeGynaudaaaGaay5waiaaw2faamaaCaaabeqaaiabiwda1iabc+caViabisda0aaaaaaaaa@5DB8@

where C¯l
 MathType@MTEF@5@5@+=feaafiart1ev1aaatCvAUfKttLearuWrP9MDH5MBPbIqV92AaeXatLxBI9gBaebbnrfifHhDYfgasaacPC6xNi=xH8viVGI8Gi=hEeeu0xXdbba9frFj0xb9qqpG0dXdb9aspeI8k8fiI+fsY=rqGqVepae9pg0db9vqaiVgFr0xfr=xfr=xc9adbaqaaeGacaGaaiaabeqaaeqabiWaaaGcbaGafm4qamKbaebadaWgaaWcbaGaemiBaWgabeaaaaa@2E87@ is given by [31]

C¯l=(4/3)0.503[1+(0.492/Pr⁡)9/16]4/9
 MathType@MTEF@5@5@+=feaafiart1ev1aaatCvAUfKttLearuWrP9MDH5MBPbIqV92AaeXatLxBI9gBaebbnrfifHhDYfgasaacPC6xNi=xI8qiVKYPFjYdHaVhbbf9v8qqaqFr0xc9vqFj0dXdbba91qpepeI8k8fiI+fsY=rqGqVepae9pg0db9vqaiVgFr0xfr=xfr=xc9adbaqaaeGacaGaaiaabeqaaeqabiWaaaGcbaGafm4qamKbaebadaWgaaWcbaGaemiBaWgabeaakiabg2da9KqbaoaalaaabaWaaeWaaeaadaWcgaqaaiabisda0aqaaiabiodaZaaaaiaawIcacaGLPaaacqaIWaamcqGGUaGlcqaI1aqncqaIWaamcqaIZaWmaeaadaWadaqaaiabigdaXiabgUcaRmaabmaabaWaaSGbaeaacqaIWaamcqGGUaGlcqaI0aancqaI5aqocqaIYaGmaeaacyGGqbaucqGGYbGCaaaacaGLOaGaayzkaaWaaWbaaeqabaGaeGyoaKJaei4la8IaeGymaeJaeGOnaydaaaGaay5waiaaw2faamaaCaaabeqaaiabisda0iabc+caViabiMda5aaaaaaaaa@4C5B@

and *D*_*o *_is the diameter of the outer cylinder (mock artery inner diameter = 0.004 m), *D*_*i *_is the diameter of the inner cylinder (collapsed stent diameter = 0.0018 m), *L *is the gap between the cylinders (0.0011 m), Ra is the Rayleigh number, and Pr = *ν*/*α *is the Prandtl number [26]. Ra is given by [31]

Ra=gβ(Ti−To)L3να
 MathType@MTEF@5@5@+=feaafiart1ev1aaatCvAUfKttLearuWrP9MDH5MBPbIqV92AaeXatLxBI9gBaebbnrfifHhDYfgasaacPC6xNi=xI8qiVKYPFjYdHaVhbbf9v8qqaqFr0xc9vqFj0dXdbba91qpepeI8k8fiI+fsY=rqGqVepae9pg0db9vqaiVgFr0xfr=xfr=xc9adbaqaaeGacaGaaiaabeqaaeqabiWaaaGcbaGaemOuaiLaemyyaeMaeyypa0tcfa4aaSaaaeaacqWGNbWziiGacqWFYoGydaqadaqaaiabdsfaunaaBaaabaGaemyAaKgabeaacqGHsislcqWGubavdaWgaaqaaiabd+gaVbqabaaacaGLOaGaayzkaaGaemitaW0aaWbaaeqabaGaeG4mamdaaaqaaiab=17aUjab=f7aHbaaaaa@409C@

where *g *is gravity (9.81 m/s^2^), *T*_*i *_is the temperature of the inner cylinder (stent temperature ~T_g _= 55°C) and *T*_*o *_is the temperature of the outer cylinder (body temperature = 37°C). Nu was calculated to be 1.8 for water and 1.6 for blood, indicating that heat transfer in the zero flow case is similar for the two fluids.

In the case of physiological flow, forced convection is the dominant heat transfer mechanism, though it depends on the flow characteristics. The flow is characterized by the Reynolds number (Re) given by [32]

Re = *U*(*D*_*o *_- *D*_*i*_)/*ν*

where *U *is the flow velocity around the collapsed stent in the mock artery at the physiological flow rate of 180 ml/min (0.3 m/s). Re was calculated to be 940 for water and 200 for blood, values that are below the critical Re of ~2000 corresponding to the onset of turbulence in a circular tube [26]. For laminar flow between two concentric cylinders with *D*_*i*_/*D*_*o *_= 0.45, Nu ≈ 6 for both fluids [33]. Based on these estimates, heat transfer in water is similar to that of blood for both the zero and non-zero flow conditions in this study.

Due to their different values of absorption coefficient (*μ*_*a*_) near the laser wavelength of 810 nm, photothermal actuation of the stent will proceed differently in water (*μ*_*a *_= 0.0021 mm^-1 ^[34]) than blood (*μ*_*a *_= 0.466 mm^-1 ^[35]). Initially, light emitted from the diffuser will reach the stent without encountering the fluid. However, as expansion continues the fluid can migrate into the pores of the inner foam cylinder. Ignoring the foam, the absorption mean free path (i.e., average distance a photon will travel before being absorbed) for water is 1/*μ*_*a *_= 476 mm; therefore, nearly all the emitted light will reach the stent throughout its expansion. For blood, the absorption mean free path is 2.1 mm, which is approximately equal to the radius of the fully expanded stent (note that scattering in blood is highly forward-directed with the average cosine of the scattering angle = 0.98 [36]). The percentage of light transmitted *T *to the expanding stent of radius *r *is given by *T *= 100exp(-*μ*_*a*_*r*); *T *= 39% for *r *= 2 mm (fully expanded). The probability that a photon will be absorbed by the blood prior to reaching the stent is higher than that for water; however, photons that are absorbed will locally heat the blood, which will, in turn, help heat the stent. For example, in the case of zero flow, the blood temperature rise due to absorption can be estimated by Δ*T *≈ *P*_*abs*_*t*/*cρV*, where *P*_*abs *_is the absorbed laser power (~100-39 = 61% of the emitted light, ignoring absorption by the stent), *t *is the laser duration in seconds, *c *and *ρ *are the specific heat and density, respectively, of blood (see Table 1), and *V *is the volume of blood in the vicinity of the stent (~2.5 × 10^-7 ^m^3 ^for a 4-mm-diameter artery). This estimate assumes the heat load is evenly distributed in the blood volume and does not include heat losses to the blood and arterial wall adjacent to the heated volume. At the maximum laser power in this study, *P*_*abs *_≈ 5 W and the blood temperature (Δ*T *+ 37°C) would reach the SMP T_g _of 55°C within 4 s. Accordingly, laser power and dye concentration in the stent may require adjustment relative to values suitable for use in water. Though laser actuation of the stent in blood is feasible in principle, experimental verification using blood is a necessary future step in the device development process.

Though *in vitro *biocompatibility studies analyzing immunostimulatory and thrombogenic properties of SMP are encouraging [17], other potential adverse effects of implantation (e.g., effect on renal function) and increased temperature due to thermal actuation are unknown and require additional study. In addition, the biocompatibility of the SMP doped with the laser-absorbing platinum dye has not been tested. If concerns were to arise based on such testing, a known biocompatible dye such as indocyanine green (ICG) could be used in place of the platinum dye. ICG is routinely administered intravenously as a contrast agent in retinal angiography, and has an absorbance band centered near the laser wavelength of 810 nm. ICG has been used to increase the laser absorption of SMP for photothermal actuation in previous work [11].

In addition to light absorption, various heating mechanisms may be employed for thermally activated SMP devices including electrical (Joule) heating [20,37,38], inductive heating [39], and heated fluid flush. For the stent application, light absorption provides a means of efficient localized heating of the dye-doped device, and the cylindrical geometry of the device is well-suited to illumination by a cylindrical light diffuser. In addition, the lack of physical connection between the light diffuser and the device enables the stent to expand freely. Inductive heating of a device doped with appropriate magnetic particles would also provide remote, localized heating, though such a device would require development of a clinically safe external source of alternating magnetic field. Localized Joule heating could be applied by embedding resistive heating elements (e.g., wires) into the device (though such elements must not interfere with shape recovery) or employing a conductive SMP composite material [20] and passing a current through the device; in either case, the stent would be physically connected to the power source and would have to be detached from the leads supplying the current following device deployment. Flowing heated saline through the delivery catheter either using a heated reservoir or embedding heating elements in the delivery catheter would expose a larger area of the vessel to elevated temperatures, which may or may not be detrimental.

Another option for SMP device deployment is to employ a low-T_g _material (≤ 37°C) such that the device spontaneously actuates at body temperature. Such a stent would not require an external heating source, and therefore would not raise the concern of thermal tissue damage. However, the modulus of the SMP at or above T_g _(~1–10 MPa) [11] is at least an order of magnitude lower than the modulus values of polymers typically used for stents in previous work [22]. Nevertheless, the fact that the SMP stent would not permanently deform if compressed (e.g., during arterial spasm), unlike some metallic stents, suggests that further study of such a design may be warranted. One group has reported complete shape recovery of prototype SMP stents designed to expand at body temperature, noting that the recovery can be controlled by varying the glass transition temperature, polymer crosslink density, and stent geometry [40].

Photothermal actuation introduces the potential to cause thermal damage to tissue and blood in the vicinity of the device. While we did not measure the temperature of the stent, the fact that the stent fully actuated indicated that it at least approached the T_g _of the stent (55°C). The temperature around the device depends on the cooling capacity of the blood and tissue, the laser power and duration, light absorption by the blood, tissue, and device, and blood flow. Thermal damage depends on the time-temperature history. For example, according to an Arrhenius damage model for arterial collagen denaturation [41], it would require several hours at a temperature of 55°C to induce irreversible damage, a timescale much longer than that needed for photothermal stent expansion. A similar model for arterial endothelial cell damage [42] indicates that cell damage would occur in less than a second at 55°C. However, standard intravascular catheter insertion and manipulation, as is used in conventional stent placement, is also known to damage the endothelial cell layer [43]. Following acute injury (mechanical or thermal) to the vessel wall and/or endothelial layer, long-term arterial recovery has been shown [44,45]. *In vivo *survival studies including histology at acute and long-term time points are required to assess the extent and tolerance of potential thermal damage caused by photothermal actuation of the SMP stent.

In practice, stents are generally placed under blood flow; however, certain other medical interventional devices, such as the MERCI Retriever for clot extraction in cerebral arteries [46], incorporate a protection mechanism to block blood flow during the intervention. The advantage of delivering the laser-activated SMP stent under a zero flow condition is that identical operating parameters (e.g., laser power and duration) can be used regardless of the flow conditions in the target vessel. In addition, by minimizing convective cooling, it reduces the amount of laser energy needed to achieve expansion.

Whether it will be clinically delivered with flow or without flow, this first prototype needs to be optimized for a controlled full actuation without causing detrimental thermal injury to the surrounding artery. Optimization could include systematically adjusting the dye concentration in the stent, possibly incorporating dye into the foam, improving the uniformity of emission along the length of the diffuser by modifying the media blasting parameters, and adjusting the foam density to enhance light scattering. In addition, the T_g _of the stent and foam can also be varied by using a different SMP material or by varying the fabrication conditions [14,15]. The T_g _should be tailored to be low enough such that the stent can be actuated at the lowest possible laser power, but it should be high enough such that the stent does not self-expand at body temperature (37°C). The current prototype was designed for use in a large vessel such as the internal carotid artery, not in the narrow cerebral vasculature. The stent pattern and dimensions would need to be scaled down to enable the device to be crimped down sufficiently for intravascular catheter delivery.

## Conclusion

We have shown the design and fabrication of a novel laser-activated stent made of shape memory polymer. In addition, we developed a fiber optic-based means of light delivery for photothermal actuation. Though further studies are required to optimize the device and assess potential thermal tissue damage, preliminary *in vitro *testing demonstrated the concept of intravascular photothermal deployment of an SMP stent.

## Competing interests

The author(s) declare that they have no competing interests.

## Authors' contributions

GB participated in the design and fabrication of the stent and artery model, design and execution of the laser deployment experiments, and writing of the manuscript. WS participated in the design and execution of the laser deployment experiments and writing of the manuscript. TSW participated in the design and fabrication of the stent. WJB participated in the design and fabrication of the stent and artery model. DLM participated in the design and supervision of the study. JH participated in the design of the stent. DJM participated in the design and supervision of the study, and design of the stent and artery model. All authors read and approved the final manuscript.

## Supplementary Material

Additional File 1 Time lapse video of SMP stent deployment in the mock artery (zero flow) as the laser power was gradually increased (see Fig. 3). Click here for file
